# Modulatory Communication Signal Performance Is Associated with a Distinct Neurogenomic State in Honey Bees

**DOI:** 10.1371/journal.pone.0006694

**Published:** 2009-08-20

**Authors:** Cédric Alaux, Nhi Duong, Stanley S. Schneider, Bruce R. Southey, Sandra Rodriguez-Zas, Gene E. Robinson

**Affiliations:** 1 Department of Entomology, University of Illinois at Urbana–Champaign, Urbana, Illinois, United States of America; 2 Institute for Genomic Biology, University of Illinois at Urbana–Champaign, Urbana, Illinois, United States of America; 3 Center for Insect Science, University of Arizona, Tucson, Arizona, United States of America; 4 Department of Biology, University of North Carolina, Charlotte, North Carolina, United States of America; 5 Department of Animal Sciences, University of Illinois at Urbana–Champaign, Urbana, Illinois, United States of America; 6 Neuroscience Program, University of Illinois at Urbana–Champaign, Urbana, Illinois, United States of America; Queen Mary University of London, United Kingdom

## Abstract

Studies of animal communication systems have revealed that the perception of a salient signal can cause large-scale changes in brain gene expression, but little is known about how communication affects the neurogenomic state of the sender. We explored this issue by studying honey bees that produce a vibratory modulatory signal. We chose this system because it represents an extreme case of animal communication; some bees perform this behavior intensively, effectively acting as communication specialists. We show large differences in patterns of brain gene expression between individuals producing vibratory signal as compared with carefully matched non-senders. Some of the differentially regulated genes have previously been implicated in the performance of other motor activities, including courtship behavior in *Drosophila melanogaster* and Parkinson's Disease in humans. Our results demonstrate for the first time a neurogenomic brain state associated with sending a communication signal and provide suggestive glimpses of molecular roots for motor control.

## Introduction

Communication is necessary for many forms of cooperative behavior. It is now well established that perception of a species-specific communication signal elicits strong changes in brain gene expression that are associated with subsequent changes in behavior. This has been shown in mammals [1]–[4], songbirds [5], cichlid [6], and swordtail fish [7] and honey bees [8], [9]. The results indicate that perception of a communication signal induces changes in brain neurogenomic states to allow animals to respond adaptively to a new situation [10]. By contrast, relatively little is known about the neurogenomic state of a sender in a communication system. Do individuals that send a communication signal also exhibit specific neurogenomic states relative to conspecifics that are not engaged in communication? Such states might reflect the tendency to engage in communication, the effects of sending a signal, or both.

Limited evidence supports the notion that sending a communication signal is associated with changes in brain gene expression. Several genes with neural functions involved in courtship signaling in *Drosophila melanogaster* have been identified [11], and the act of singing induces *EGR-1* and synelfin in the song production areas of the zebra finch (*Taeniopygia guttata*) brain [12], [13]. However, genomic analyses of communication signal senders has not kept apace with analyses of receivers, and it is not known whether sending a communication signal is associated with comparably large-scale effects on brain gene expression.

We examined this issue by studying brain gene expression associated with “vibration signal” communication in honey bees. Honey bee colonies are composed of tens of thousands of individuals and rely on diverse chemical, visual and mechanosensory communication signals to coordinate activity to changing conditions. Vibration signal communication involves one bee grasping another with its forelegs and rapidly vibrating its body dorso-ventrally in a highly stereotypical manner for 1–2 s (see video S1 - the bee tagged as red 51 begins performing vibration signals at 9 seconds). The vibration signal has a modulatory effect in a variety of contexts. Recipients of the vibration signal increase their level of task performance, enhancing many different activities, including foraging, brood care, swarming, house-hunting, queen behavior, and queen rearing behavior by worker bees [14]. Bees engaged in vibration signal performance thus apparently are able to perceive changes in colony needs and modulate the activity of their nestmates accordingly [15].

We chose this system for our analysis, rather than another form of mechanosensory communication, the more famous “dance language” of the honey bee [16] because vibration signal communication involves a cadre of individuals that perform this behavior intensively over an extended period of time, making it particularly appropriate for genomic analysis. Dance communication occurs briefly, faster than the scale of gene transcription, making it more challenging to directly link changes in gene expression with the production of dance communication (see [17] for a study that indirectly links changes in gene expression with the production of dance communication.) Only a subset (<15%) of a colony's bees ever perform vibration communication signaling during their lifetimes [14]. These senders engage in bouts that involve contacting 20 or more bees per minute and last from several minutes to over an hour. Due to the presence of vibration signal “specialists” the vibration signal represents a good model to ask whether individuals that are sending a communication signal exhibit a unique neurogenomic state, manifested as a specific pattern of brain gene expression.

We used microarray analysis to compare brain gene expression between bees that performed vibration signaling persistently (V+) and carefully matched bees that never performed it (V−).

## Results and Discussion

### Vibration signal senders have a specific brain gene expression profile

**Figure 1:**
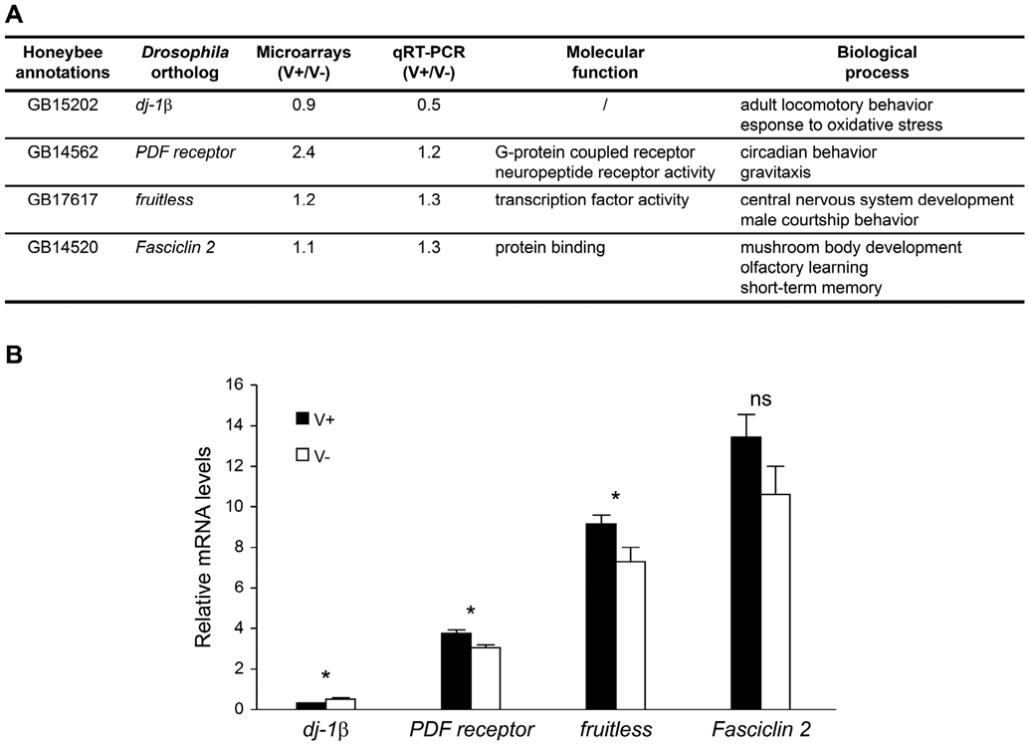
Genes differentially expressed between vibration signal performers and non-performers. A. Description of the 4 genes analyzed, chosen from among the 918 differentially expressed between V+ and V− bees functions based on Gene Ontology information for *Drosophila melanogaster* orthologs. B. Brain mRNA levels for these 4 genes. n = 7 individuals/group. qPCR data were normalized to expression levels of *eIF3-S8*. Significant differences were determined using a Wilcoxon signed rank test (*p<0.05, ns = not significant). Means±s.e. are shown. The four genes were chosen because of their functions in *Drosophila*, which can be linked plausibly to vibratory communication signal in bees. Vibrating bees display a high rhythmic locomotion rate, and *dj-1β*, PDF receptor and fruitless are involved in locomotory and rhythmic behavior [31]–[35]. They also need to assess and memorize the colony needs and *Fasciclin 2* is involved in olfactory learning and mushroom body development [36], [37]. Differences in expression were detected in 3 out of 4 genes with qPCR; these results are not inconsistent with expectations from the False Discovery Rate used in this study for analysis of microarray results.

We used an oligonucleotide microarray based on gene predictions and annotation from the honey bee genome sequencing project [18]. A total of 903 genes were found to be differentially expressed in the brains of V+ and V− bees (False Discovery Rate <0.05, p<0.005; gene list is in Table S1; see Fig. 1 for qRT-PCR confirmation of a few of the microarray results). This was a surprisingly large number of genes, given that V+ and V− bees were all foragers, matched for behavior, age, genotype, and foraging experience (Table S2). 412 genes were upregulated in V+ compared to V− bees and 491 were downregulated. For comparison, experiments reported elsewhere using the same microarray and with comparable statistical power showed only 58 genes differentially expressed in the brain between foragers belonging to African and European honey bee subspecies (unpublished data) and 1396 between young bees that work in the hive (brood care) and old bees that forage [8]. The magnitude of the differences in expression for the 903 genes ranged from 5 to 254%, which is comparable to values reported in other studies of brain gene expression [8], [19]. The large number of genes associated with vibration signaling suggests that senders of other forms of communication signals, even those more fleeting than vibration signal, may also display a specific neurogenomic state.

### Brain gene expression profile of vibration signal senders suggests arousal

The performance of vibratory communication leads to increased task performance, suggesting that vibration communication is a response to the perception of specific colony needs. If so, bees specializing on vibration communication might be in a state of heightened arousal, associated with their perception of these needs. To explore this idea, we compared the brain gene expression profile of V+ bees with genomic profiles from another study that were induced by perception of pheromone signals (causing arousal in receivers).

We first compared the V+ genomic profile with bees that show heightened arousal due to exposure to alarm pheromone (unpublished data). Bees that are aroused by alarm pheromone visually search for intruders in the vicinity of the hive, and perception of movement stimulates stinging [20]. Expression patterns of V+ bees were similar to those exposed to alarm pheromone (Table 1). A larger proportion of genes upregulated by alarm pheromone were upregulated in V+ bees, and a larger proportion of genes downregulated by alarm pheromone were downregulated in V+ bees; this distribution was highly significant (p<0.001, χ^2^ = 11.21).

**Table 1:** Vibration signal performance and arousal: overlap of genes regulated in vibration signal performers and bees exposed to different pheromones.

	V+↑ (412)	V+↓ (491)	χ2 and p-values
Alarm pheromone ↑ (237)	22 ^[32%]^	12 ^[17%]^	χ2 = 10.65
Alarm pheromone ↓ (201)	8 ^[12%]^	27 ^[39%]^	p<0.005
Queen mandibular pheromone ↑ (374)	15 ^[21%]^	25 ^[35%]^	χ2 = 7.11
Queen mandibular pheromone ↓ (323)	23 ^[32%]^	9 ^[13%]^	p<0.01
Brood pheromone ↑ (122)	14 ^[27%]^	8 ^[16%]^	χ2 = 1.10
Brood pheromone ↓ (106)	13 ^[26%]^	16 ^[32%]^	p = 0.29

Number and direction of expression (indicated by arrows) of genes that are differentially regulated in V+ and V− bees and also by one or more pheromone. Gene expression data were taken from [9] for queen mandibular pheromone and from [8] for brood pheromone. Numbers in parentheses are the total number of genes from each experiment. Numbers in brackets are the percentages of genes regulated in V+ bees that are up- or downregulated by pheromones. Chi-square tests with Yates correction were performed.

By contrast, the V+ expression pattern was likely opposite to the pattern caused by exposure to queen mandibular pheromone [9]. Queen pheromone decreases dopamine signaling, which depresses motor activity and learning and memory [21]–[23], arguably related to arousal. A larger proportion of genes upregulated by queen pheromone were downregulated in V+ bees, and a larger proportion of genes downregulated by queen pheromone were upregulated in V+ bees (Table 1); this distribution was highly significant (p<0.005, χ^2^ = 8.19). We also compared the brain gene expression profile of V+ bees to bees exposed to brood pheromone [8], which is not known to affect arousal. There was no specific pattern of association. The results of these comparisons suggest that V+ bees display a brain gene expression profile associated with heightened arousal.

### Brain gene expression profile of vibration signal senders suggests connections to motor behavior in flies and humans

**Figure 2:**
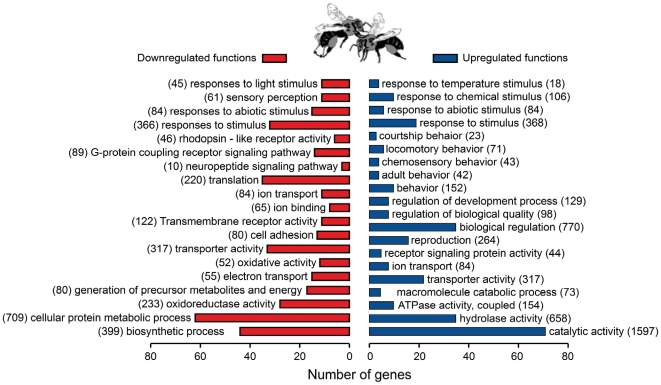
Functional analysis of genes associated with modulatory communication signal performance in honey bees. Gene Ontology molecular process and biological function categories that were significantly enriched in the gene sets down- and upregulated in V+ compared to V− bees (p<0.05). Categories are non-mutually exclusive. For each enriched category, the total number of genes with fly orthologs expressed on the microarray is given in parenthesis and the number of genes differentially expressed between V+ and V− bees is given by the x-axis.

Functional analysis of the gene set differentially expressed between V+ and V− bees using Gene Ontology (GO) revealed a set of molecular function and biological process categories that were significantly overrepresented (Fig. 2). These include subsets of genes involved in “response to chemical stimulus” and “locomotory behavior,” which were significantly overrepresented in the V+ upregulated gene set. This result is consistent with the observation that V+ bees walk extensively through the hive when engaged in signaling [24].

Another GO category showing enrichment in V+ bees was “courtship” (in *Drosophila melanogaster*), which also involves vibratory communication. *fruitless*, a key gene in *Drosophila* courtship, is differentially expressed between V+ and V− bees (Fig. 1). These results suggest that genes shown to be involved in locomotion or behavioral signaling in *Drosophila* play similar roles in bees.

A surprising finding was that 5 genes implicated in Parkinson's Disease (PD) are also associated with vibration signal performance (Table 2). We then looked whether this pattern of expression for these 5 genes was also seen in other honey bee brain microarray experiments. Searching the lists of differentially expressed genes from twelve honey bee brain microarray experiments published to date (see Methods), revealed that the only condition that affects more than half of these genes (*DJ-1*, *SUMO-1*, *UBC7*) is manganese treatment [25] (Table 2). Manganese is known to cause symptoms in humans similar to PD [26]. These results suggest that genes involved in motor control in humans are also involved in vibratory communication in honey bees. Proving this would of course require additional experimentation.

**Table 2:** Genes involved in vibration signaling in bees and Parkinson's disease in Human.

Honey bee gene^#^	Honey bee gene name	*Drosophila* ortholog	Human ortholog	V+/V−	Parkinson's disease characteristics
GB15202^*^	*/*	*dj-1β*	*DJ-1*	↓	Mutation in *DJ-1* causes autosomal recessive early-onset parkinsonism [38]
GB19379^*^	*/*	*/*	*SUMO-1*	↓	*DJ-1* interacts with *SUMO-1* to be fully active [39]
GB18477^*^	*/*	*courtless*	*UBC7*	↓	*UBC7* interacts with *Parkin* [40] responsible for autosomal recessive early-onset parkinsonism [41]
GB30031	*Dop1*	*DopR*	*/*	↓	Low level of dopamine [42]
GB16377	*Eaat*	*/*	*EAAT2*	↑	Increased glutamate signaling [43]

Downregulation of *dj-1β* has been confirmed by qRT-PCR (Fig. 1). ^*^indicates genes also regulated by manganese treatment in bees [25].

### Conclusions

Functional hypotheses about animal signaling usually refer to the mental states of both sender and recipient [27], which currently are related to neurophysiological activity [28]. Together with earlier research [10], our results show that animal communication also is characterized by distinct neurogenomic states in the brains of both senders and receivers. In senders, the global neurogenomic state at the whole-brain level might reflect the effects of sending a signal on brain gene expression or the tendency to engage in communication, or both. It now will be interesting to explore how sender and receiver are coupled at the level of brain gene expression.

## Materials and Methods

### Sample collection

Experiments were performed at the University of North Carolina, USA, with colonies of *Apis mellifera* bees that contained a mixture of different subspecies, primarily *A.m. ligustica*. Colonies were maintained according to standard commercial procedures. We examined brain gene expression patterns in individual workers for which we determined lifetime behavioral profiles for vibration signal and foraging activity. Each worker belonged to one of three patrilines (designated A, B and C), which were derived from three separate queens each instrumentally inseminated with the semen of a different unrelated drone. The resulting workers within each patriline shared, on average, 75% of their genome, which standardized the genetic background against which differences in brain gene expression patterns were assessed. Each of the inseminated queens headed a separate colony. Honeycomb frames containing older (“capped”) brood from each of the three colonies were placed inside separate, pre-labeled nylon-mesh cages and transferred to an incubator (32.5°C; 50% RH). We collected 1000 newly emerged workers from each patriline and marked them on the thorax with colored numbered plastic tags for individual identification (Opalithplättchen, Chr. Graze, Endersbach, Germany). Workers were marked within 12 h of adult eclosion so that their exact age (in days) was known throughout the study. All 1000 workers collected from each patriline were tagged within a two-day period. Each group of tagged workers was added to a separate colony housed in a glass-wall four-frame observation hive headed by an unrelated, naturally mated queen. The observation colonies were labeled A, B and C (colony A contained patriline A, etc.) and matched for areas of honeycomb containing brood and food, and population size. The observation hives were set up simultaneously and each was fed sucrose solution (50% by volume) *ad libitum* throughout the study to help equalize foraging success and food reserves among colonies. The sides of the observation hives were composed of plexiglass sheets with hinged access ports through which workers could be collected. The colonies were maintained for 5 weeks, by the end of which time the vast majority of tagged workers had died.

To generate lifetime behavioral profiles, each colony was monitored continuously by two randomly-assigned assistants from 0800 to 1700–1800 h every day throughout the 5-week study period starting at 2 days of age for the tagged bees, for a total of 330 h of observation/colony. Throughout each day, we recorded the identity and age of every tagged bee observed to perform vibration signals (see video S1), waggle dances (an indication of successful foraging), and carry pollen loads (another indication of successful foraging). Subsequently, we determined the total number of days that each tagged individual performed the different activities (Table S2).

At the end of the study period, we removed tagged individuals that were immediately performing vibration signals and had vibrated on at least three days during their lifetimes (V+ bees). For each V+ worker collected, we collected within 5 min a control bee (V-) that was of the same age and patriline and had comparable levels of foraging experience, but which had never been observed performing vibration signals during its lifetime. All collected workers were immediately flash frozen in liquid nitrogen and stored in pre-labeled vials at −80°C until brain dissection. All bees, V+ and V-, were of foraging age. A total of 14 age-matched V+/V− pairs were collected from the three colonies (Table S2). The number of individuals that performed waggle dances or carried pollen loads was identical for the V+ and V− bees, suggesting the two groups experienced similar levels of foraging success. The observed patterns of brain gene expression are therefore unlikely to have arisen from differences in foraging experience, but rather reflect differences in signaling activity per se. Further details of the methodology are given in [29].

### Microarrays

Bee heads were partially lyophilized to facilitate brain dissection. Individual brains were homogenized in 500 µl of Trizol (Invitrogen Life Technologies). The mixture was incubated for 5 min and then 100 µl of water and 100 µl of Chloroform were added and allowed to incubate for 3 min. The solution was centrifuged at 12,000 g (4°C) for 15 min. The aqueous phase was mixed with an equal volume of 70% ethanol and transferred into a Qiagen RNeasy column. RNA extraction was carried out as indicated in the Qiagen RNeasy kit for total RNA with on-column DNase I treatment (Qiagen, Valencia, CA). To quantify gene expression from individual brains, RNA (500 ng) was amplified with the Amino Allyl MessageAmp™ II aRNA Amplifcation kit (Ambion, Austin, TX), according to the kit instructions. *in vitro* transcription proceeded with an incubation time of 4 h at 37° C. 2.5 µg aRNA was used for microarray hybridization. Dye coupling and labelled aRNA cleanup was carried out with the Amino Allyl MessageAmp™ II aRNA Amplifcation kit. Sample was dried down and resuspended in 4.5 µl Coupling buffer (0.1 M carbonate buffer pH 9). At the end of the procedure an equal volume of 2X hybridization buffer was added.

Each pair of bees (n = 14) was directly compared on the same microarrays (with dye swap) giving a total of 28 arrays. Slides were passed quickly through steam and placed in a UV linker at 6000×100 µJ/cm^2^. Before pre-hybridization, slides were plunged in 0.2% SDS and immediately shaken vigorously for 2 min. They were then washed twice in distilled water, transferred to 95% ethanol for 15 sec, and dried at 2000 rpm for 3 min. For hybridization, slides were incubated at 42°C in a Coplin jar for ∼1.5 h, then washed in distilled water twice and isopraponol and dried at 2000 rpm for 3 min. Samples were incubated at 95–100°C for 3 min and then kept at 55°C until applied to the microarray slides. 75–80 µl of sample was applied on the slides and slides incubated for 18 h at 42°C. Excess sample was removed by a series of 4 washes with shaking (75 rpm): 10 min in 1X SSC, 0.2% SDS; 10 min 0.1X SSC, 0.2% SDS; 15 min in 0.1X SSC (twice). Dyes used to label each sample were reversed in half of the replicates to control for dye-by-gene interactions. Slides were scanned using an Axon 4000B scanner, and images analyzed with GENEPIX software (Agilent Technologies, Santa Clara, CA).

Spots were removed from analysis if flagged by the GENEPIX software or if the fluorescence intensity was less than the median intensity of the negative control spots. A Loess transformation was performed using Beehive (http://stagbeetle.animal.uiuc.edu/Beehive) to normalize expression intensities. A linear mixed effects model implemented using Restricted Maximum Likelihood was used to analyze the normalized log2 transformed fluorescence intensities for each gene, accounting for the effects of dye, treatment, bee and microarray. Treatment effects were evaluated with F-test statistics and the p-values were adjusted for multiple testing using a False Discovery Rate criterion. Filtering of genes abundantly expressed in hypopharyngeal glands (a potential source of tissue contamination in brain samples) was done as in [8]. Genes that showed a fold change lower than 5% were excluded.

### mRNA Quantification by Real-Time qRT-PCR

Confirmation of some of the results obtained from microarray analysis was performed with real-time quantitative RT-PCR for 7 individual brains/group used for the microarrays. Expression levels were measured for *dj-1β*, *PDF receptor*, *fruitless* and *Fasciclin 2* with an ABI Prism 7900 sequence detector and the SYBR green detection method (Applied Biosystems). *eIF3-S8*, a housekeeping gene that did not vary in expression levels on the microarrays or in the quantitative RT-PCR (Wilcoxon signed rank test: p>0.3), was used as loading control [8]. The sequences for the primers used are given below. Results are consistent with the microarray results (Fig. 1).

Primer sequences (5′ to 3′) were *dj-1β* forward: CCTACTGCATTAAAGGCTCATGGT, reverse: TTGATCCTTCATTGCAGGATAAGA; *PDF receptor* forward: CCGGTCTGGGACTCGTTACTC, reverse: CGTATGGGCATCTTTGTTTGG; *fruitless* forward: ACATGCGGCTGACCTTTGAC, reverse: CGTGGTAGTGGTTCCTGATGTG; *Fasciclin 2* forward: ACTCGAGAACAGTGGCGATGA, reverse: GATCTGAGGGACTGGCTGATG; *eIF3-S8* forward: TGAGTGTCTGCTATGGATTGCAA, reverse: TCGCGGCTCGTGGTAAA.

### Functional analysis

Gene Ontology (GO) enrichment analysis was performed only with genes differentially expressed between V+ and V− bees that also had clear *Drosophila melanogaster* orthologs. Enrichment was determined using GOToolBox (http://burgundy.cmmt.ubc.ca/GOToolBox/) with a hypergeometric test followed by the Benjamini and Hochberg False Discovery Rate adjustment for multiple testing (GO categories at p<0.05 shown) [30]. For this analysis, the reference gene set corresponds to the total number of genes with fly orthologs shown to be expressed on the microarray. The honey bee brain microarray experiments used for comparative analysis are: E-MEXP-24, E-MEXP-79, E-MEXP-80, E-TABM-149, E- TABM -150, E- TABM -151, E-MEXP-252, E-MEXP-262, E-MEXP-512, E-MEXP-699, E-MEXP-1044, E-MEXP-1552) (http://www.ebi.ac.uk/microarray-as/ae/).

## Supporting Information

Table S1 List of genes differentially expressed in the brains of V+ and V− bees. Corresponding Drosophila and Human orthologs and log2 (V+/V− ratio) expression values are shown. Genes differentially regulated in V+ and V− bees and also up- or downregulated by one or more pheromone are indicated.
(0.14 MB XLS)

Table S2 V+ and V− bees matched for behavioral category, age, genotype, and foraging experience.
(0.04 MB DOC)

Video S1 Bee (n°51, red tag) performing vibration signal. The bee bearing the tag Red 51 begins performing vibration signals at 9 sec.
(9.37 MB MPG)
