# Supplementary material for: Modulatory Communication Signal Performance Is Associated with a Distinct Neurogenomic State in Honey Bees
Source: PLoS One. 2009 Aug 20;4(8):e6694. doi: 10.1371/journal.pone.0006694 (PMC2725773; doi:10.1371/journal.pone.0006694)
Supplement: Table S2 — V+ and V− bees matched for behavioral category, age, genotype, and foraging experience. (0.04 MB DOC) [file pone.0006694.s002.doc]

**Table S2**: **V+ and V- bees matched for behavioral category, age, genotype, and foraging experience.**

| **Patriline** | **Bee Id (V+)** | **Age** | **History** | **Bee Id (V-)** | **Age** | **History** |
| --- | --- | --- | --- | --- | --- | --- |
| A | 3+ | 21 | 4V 1W 0P | 3- | 21 | 0V 0W 0P |
| A | 25+ | 25 | 3V 0W 0P | 25- | 25 | 0V 0W 0P |
| A | 35+ | 29 | 3V 0W 0P | 35- | 29 | 0V 0W 1P |
| B | 10+ | 19 | 4V 0W 0P | 10- | 19 | 0V 0W 0P |
| B | 17+ | 20 | 6V 0W 1P | 17- | 20 | 0V 0W 1P |
| B | 19+ | 21 | 3V 0W 0P | 19- | 21 | 0V 0W 1P |
| B | 24+ | 22 | 7V 1W 1P | 24- | 22 | 0V 0W 1P |
| B | 37+ | 28 | 4V 0W 1P | 37- | 28 | 0V 0W 1P |
| B | 43+ | 30 | 5V 0W 3P | 43- | 29 | 0V 0W 2P |
| C | 16+ | 22 | 3V 0W 0P | 16- | 22 | 0V 0W 0P |
| C | 39+ | 26 | 5V 0W 1P | 39- | 26 | 0V 0W 0P |
| C | 44+ | 28 | 3V 0W 0P | 44- | 28 | 0V 0W 0P |
| C | 46+ | 29 | 4V 0W 0P | 46- | 29 | 0V 0W 0P |
| C | 47+ | 30 | 3V 0W 0P | 47- | 30 | 0V 0W 0P |

*Genotype* = patriline. Each patriline was derived from a queen instrumentally inseminated with semen from a single male. Workers from each patriline were maintained in separate glass-walled observation colonies. *Bee Id* = code for individual bee identification. We recorded all incidences of vibration signal performance, waggle dance behavior (an indicator of foraging status), and pollen collection (another indicator of foraging status) for the tagged bees and subsequently determined the total number of days each activity was observed for every focal individual during its lifetime. *V+* = tagged individuals that were performing vibration signals at the moment of collection and which had produced signals on at least 3 preceding days. *V-* = tagged bees of the same patriline as the V+ counterpart that were never observed to perform vibration signals or waggle dances during their lifetimes. *History* = lifetime behavioral profile: *V* = number of days bees were observed to perform vibration signals; *W* = number of days bees were observed performing waggle dances; *P* = number of days bees were observed with pollen on legs.
